# Are probiotics a feasible intervention for prevention of diarrhoea in the developing world?

**DOI:** 10.1186/1757-4749-2-10

**Published:** 2010-08-29

**Authors:** Neerja Hajela, Gopinath B Nair, Nirmal K Ganguly

**Affiliations:** 1 Yakult Danone India Private Limited, New Delhi - 110020, India; 2 National Institute of Cholera and Enteric Diseases, Kolkata - 700010, India; 3 National Institute of Immunology, New Delhi - 110 067, India

## Abstract

With more than 1.4 million of the 9 million child deaths being attributed to diarrhoea in 2008 and 49% of them occurring in five countries namely, India, Nigeria, Democratic Republic of the Congo, Pakistan and China, there is an urgent need for intervention to prevent and control diarrhoeal diseases. Of the various interventions, probiotics offer immense potential. The past decade has witnessed the validation of their utility for the prevention, treatment and management of a variety of infective and non infective disorders. The most investigated field continues to remain infectious diarrhoea and compelling evidence comes from randomized placebo controlled trials. While results from these studies are encouraging most of them reflect the outcomes of the developed world. Developing countries like India continue to struggle with nutritional and health challenges and bear the greatest burden of diarrhoea. A paucity of data from the developing countries limits the definite recommendation of probiotics. In these countries curd, often confused for a probiotic, is practiced as an integral part of the culture. While the nutritional benefits of these products cannot be understated, it is still uncertain whether these products can be classified as a probiotic. The emergence of probiotic foods which are scientifically validated for their efficacy and impart defined health benefits offer an excellent opportunity to improve public health. A recent randomized controlled trial conducted by the National Institute of Cholera and Enteric Diseases in Kolkata, India demonstrated a protective efficacy of 14% in preventing diarrhoea among children who received a probiotic. For the developing world however the vision for probiotics would mean a fundamental change in perception and developing a well planned strategy to allow interventions like probiotics to permeate to impoverished settings, where the assault of micro organisms is on a daily basis. This would mean that probiotics are ingrained into the public health system without being seen as a medicine.

## 

Dr. Elie Metchnikoff, a Russian laureate, is credited with introducing the concept that live microorganisms are beneficial for health. In 1965, this concept was formalized by introduction of the term "Probiotics" by Lilly and Stilwell to define growth promoting factors produced by microorganisms. Although the concept of probiotics goes back to the Vedic times, the science has taken a giant leap only in the recent past. A substantial part of this new-found interest stems from the unprecedented advancement in our understanding of the role of the intestinal microbiota. A few decades ago, only 400 bacterial communities were reported to colonize the distal human gut. The development of metagenomics supported by high throughput sequencing has launched this number to about 1000 communities [1]. More importantly, there is substantial progress in understanding the function, autochthony and microbial ecology of this complex ecosystem. The intestinal microbiota today is thought to be an organ that works like a well orchestrated symphony in health and can be manipulated to improve health, when in disease.

These insights have led to escalation in probiotic research resulting in significant expansion of the probiotic arena especially in the Western world with more than 500 new stock keeping units globally in the probiotic foods and beverages sector [2]. Research Institutes solely working on probiotics have mushroomed in many parts of the developed world. There is however a distinct difference between the Western and the Eastern perspective on the use of probiotics. While in the West they are considered a revolution and viewed as a means for maintaining a balanced gut flora geared towards optimum health, in Asia and the Orient probiotics continue to remain a scientific curiosity, a clinicians' dilemma and a confusing entity to the general public as to what makes them different from traditional foods.

Probiotics find use in a variety of diseases including diarrhoea and although several products are becoming increasingly available they are not commonly recommended. The possible use of probiotics as a public health strategy such as improved sanitation or delivery of safe drinking water is not contemplated and the rural and urban poor areas have largely been neglected. Ironically, it is this group that has the heaviest burden of diarrhoea, acute respiratory infections and a host of other infectious diseases [3]. Poverty is a defining factor in the high burden of diarrhoea and also a defining factor for the exclusion of health benefits derived from interventions like probiotics. In this editorial, we debate on how probiotics should be used as a public health strategy to prevent diarrhoea in populations where the need is the greatest.

The definition of probiotics has been widely debated, but in 2001 FAO and WHO defined them as "Live microorganisms which when administered in adequate amounts confer a health benefit on the host" [4]. This universally accepted definition implies that a probiotic strain unless protected by a capsule should be intrinsically resistant to low pH, bile and pancreatic enzymes to ensure GI transit in numbers adequate enough to elicit a defined benefit to the host. It was then recognized that the concept of probiotics is essentially to improve host health by modifying the composition of the intestinal microbiota. The recent advent of powerful molecular techniques have made it possible to monitor changes in the gut micro biota following probiotic administration thereby enabling better understanding of their function. This has also helped to recognize the fact that probiotics offer remarkable potential for the prevention and management of various infective and non infective disorders. Scientific evidence points to the fact that the ability of a probiotic bacteria to confer a health effect largely depends on the particular strain being used [5]. There has thus been a resurgence of interest about the strain specific benefits of probiotics and clinical research is quickly accumulating to support the evidence for their use. The scope and scale of the potential of probiotics is staggering and the full spectrum of their benefit to human health is still being investigated.

With the realization of what it takes to classify as a true probiotic it remains uncertain whether curd and other dairy products are actually probiotic. According to The Prevention of Food Adulteration Act and Rules, 1955 [6], curd is defined as a product obtained from souring of boiled or pasteurized milk naturally, by harmless lactic acid bacteria or other bacterial cultures [A. 11.02.04 of Appendix B, Definitions and Standards of quality] whereas Yoghurt is obtained by lactic acid fermentation of milk by *Lactobacillus delbreuckii *ssp*. bulgaricus *and *Streptococcus thermophilus*. It may also contain *Bifidobacterium bifidus *and *Lactobacillus acidophilus *and other cultures of harmless lactic acid producing bacteria which if added must be declared [A. 11.02.17 of Appendix B, Definitions and Standards of quality]. While one cannot undermine the nutritional benefits of these products, it still remains to be determined whether these products contain organisms that are defined in terms of number, viable at the target site and proven for their ability to impart strain specific benefits. Therefore the emergence of probiotic foods which are scientifically validated for their efficacy and impart defined health benefits offer an excellent opportunity to improve public health and the category is receiving far more attention than what was originally conceptualized. India with its growing awareness and need for preventive medicine is also witnessing the entry of a surge of probiotic foods which are rapidly finding their way onto the market shelves. But would these products really find use in a developing country like India still remains to be seen.

Around 1.4 million of the 9 million child deaths in 2008 were due to diarrhoea with 49% of the deaths occurring in five countries namely India, Nigeria, Democratic Republic of the Congo, Pakistan and China [3,7]. In 2008, diarrhoea killed more children than AIDS, malaria and measles combined. Progress has been made in some areas of diarrhoea prevention. These include vitamin A supplementation, immunization, access to safe drinking water and exclusive breast feeding [7,8]. However, current estimates show that 2.5 billion people still lack access to improved sanitation facilities and 1 billion lack access to improved drinking-water sources. The other attendant effects of diarrhoea include underweight or stunting which causes about 20% of all mortality of children younger than 5 years of age [9]. Unless, these statistics improve diarrhoea will continue to remain a formidable but largely neglected problem in many parts of the developing world.

The rationale for using probiotics in acute infectious diarrhoea is based on the assumption that they act against intestinal pathogens and possible mechanisms include the synthesis of antimicrobial substances, competitive inhibition of adhesion of pathogens, modification of toxin and non toxin receptors and stimulation of non specific and specific immune responses to pathogens. While research using probiotics has extended to a vast array of diseases, the most investigated field continues to remain infectious diarrhoea and compelling evidence comes from randomized placebo controlled trials. Data extrapolated from a large body of studies that include systemic reviews [10-13] meta analysis [14-17], open label studies [18,19], multicenter trials [20] testing the efficacy of probiotics in preventing diarrhoea concluded that besides demonstrating a good safety profile probiotics significantly reduced the duration and frequency of acute diarrhoea. A meta-analysis of acute pediatric diarrhoea concluded that there was significant data for probiotic based reduction of diarrhoea duration, treatment failure and prevention [21]. Different strains of Lactobacilli have also shown benefit in reducing the duration of rotaviral shedding; an observation that has favorable epidemiologic implications [19,22]. A Cochrane review suggests that probiotics may appear to be a useful adjunct to rehydration therapy when managing both adults and children [10]. The beneficial effects of probiotics however are strain dependent, dose dependent, greater for doses of more than 10^10 ^cfu, significant in people with viral gastroenteritis and more evident when treatment with probiotics is initiated early in the course of the disease [23]. It is more difficult to assess benefit in adults due to the range of strains, products studied and a lack of identification of the pathogens involved. However, recently there has been convincing evidence for probiotic reduction of the risk of antibiotic associated diarrhoea and conclude potential for Lactobacillus species and *Saccharomyces boulardii *probiotics [12,14,16,21,24]. These findings were substantiated by a careful meta analysis by D' Souza *et al *[24] that found probiotics more effective than placebo.

Although the results from these studies appear to be encouraging, they come with one major disadvantage - most of them reflect the outcomes of the developed world. Therefore, while they have resulted in changing the face of the health map in the Western world, the developing world continues to grapple because of a paucity of data and the concept remains unproven and blurred. The goal is to set the stage. A leap forward would mean taking cue from the findings of the developed world that could herald probiotic usage in a country like India which continues to struggle with nutritional and health challenges and hence bears the highest burden of diarrhoea [3]. While a number of studies evaluating the role of probiotics in preventing diarrhoea have been conducted in the developing world they have failed to document any significant benefit [25-27]. Most of the trials that have been conducted in India to evaluate the prophylactic and therapeutic role of various probiotic strains in diarrhoea have focused on children but unfortunately have shown mixed results [28-31]. While some have shown benefit others have demonstrated no visible difference. This has limited their definite recommendation and therefore calls for design of studies that would allow definite use of a particular probiotic strain. This more so given that a substantial pediatric hospital admission is due to acute watery diarrhoea which strain the hospital resources and significantly impact on the economic burden of the country. Probiotics in the form of a ubiquitous, simple, safe intervention, if found effective in limiting the duration of diarrhoea, would be a welcome addition to the strategy of containment of diarrhoea related complications. A recent community based trial on more than 3000 children at the National Institute of Cholera and Enteric Diseases, Kolkata where the use of a probiotic strain *Lactobacillus casei *strain Shirota showed a protective efficacy of 14% in preventing acute diarrhoea brings a ray of hope [32].

However in countries where curd, often confused for a probiotic, forms an integral part of the culture, the concept is bound to be met with much resistance and skepticism. It is thus about entering a new, unchartered territory. Unfortunately compelling scientific evidence from the West is not enough to substantiate belief about the health benefits of probiotics when it comes to key regulators and policy makers. This is with good reason given that poly microbial diarrheal infections are common in these settings. It is therefore not surprising that while the growing scientific developments in the area are setting public, industry and scientific research agendas on one hand, on the other it has led to a wave of concern raising a number of critical questions which are fundamentally different from those raised in the past. Key concerns are: can experts be believed, do probiotics do what they say, for how long does one consume them to get a health benefit, do they provide any additional benefit than that conferred by regular consumption of curd, are they scientifically validated for their efficacy, are they affordable, what is the interpretation of risk, disease and illness, regulation and public health impact. A frequently asked question relates to the dose of a probiotic that needs to be ingested to trigger an effect. Often key debate on the use of probiotics centers on their ability to colonize the Indian gut, which because of multiple pathogen onslaught is microbiologically more hostile and is therefore considered to be different from that of the Western counterpart. Also whether a probiotic would confer a similar response in both the settings. A key goal of probiotic science would thus be to understand and target particular biomarkers and end points for the disease and the specific strains that act upon this. Put simply the scientific challenge is about identifying the strain specific benefits.

From a regulatory standpoint also this area is highly unregulated. Looking at the global scenario, probiotics fall into a grey regulatory area because regulations for probiotics are nonexistent in most countries. Whatever the regulations they differ from country to country and within the same country the way the product is regulated. Japan is by far the most advanced in terms of regulations with the Japanese Ministry of Health, Labour and Welfare (MHLW) having set up "Foods for Specified Health Use" (FOSHU) in 1991 as a regulatory system to approve the statements made on food labels [33]. In India there is no regulatory framework for probiotics in food, however the category is expected to witness rapid growth in the coming years. In the absence of any regulatory standards and guidelines there would always be a possibility of products that are unreliable in content and contain strains whose scientific efficacy has not been validated. Hence it becomes imperative that these products are standardized and fulfill the desired effect through evidence based studies which is what prompted the Indian Council of Medical Research (ICMR) and Department of Biotechnology (DBT) to establish a set of guidelines that would ensure product safety, quality, reliability and level playing for all companies introducing and producing probiotic products. These guidelines when implemented would have a provision for assessment of efficacy; safety and health claims made by the probiotic foods that are being launched [34]. Despite this the main concern that continues to surround most critics is whether probiotics represent just another trend and hence the question often is where is the dividing line - in other words just how much science is enough, what are the implications for their use and should recommendations be made to the public. The list of questions and more questions is endless and the application of probiotics continues to remain a paradox. Thus despite the fact that probiotics represent a unique perspective for improvement of health the optimism that probiotics accrue in terms of benefit is counter balanced by a series of 'ifs' and 'buts' limiting their definite recommendation for a developing country like India.

For the developing world, therefore the vision for probiotics would mean a fundamental change in perception. It means developing a diet based intervention strategy and formulating foods tailored to meet the specific health needs. However a developing country often faces challenges from changing demographic profiles, sedentary lifestyles, higher disposable income on one hand to deepening poverty on the other. It therefore becomes important that the probiotic claim acts as a significant signal of value appealing to the consumers growing interest in the link between their diet and their health justifying a significant price premium. It remains uncertain though whether probiotics are seen as an intervention for healthy individuals or those who are ill and have a medical condition or for both. This important distinction therefore needs to be made.

While the probiotic revolution promises to take health to new heights or as the scientists openly say to new frontiers, the most effective direction to take continues to be the subject of much debate which will lead to arguments for and arguments against and will include that some of the major issues such as efficacy in the given scenario, and acceptable outcomes should be reached. This editorial begs the question whether probiotics will contribute to public health goals or simply be seen as addressing a narrow range of unsubstantiated individual effects. The challenge in the next few years would be to develop a well planned strategy to allow interventions like probiotics to permeate to impoverished settings where the assault of microorganisms, pathogenic or otherwise, is on a daily basis. Such settings are very different from the sanitized West or the affluent sections in the East. The challenge for a probiotic to find its way to the masses would be governed by costs, compliance, and concept on how to get this ingrained into the public health system without being seen as a medicine.

## Competing interests

GBN and NKG declare that they have no competing interest.

NH is a Biotechnologist and the Head of Science, Yakult Danone India Pvt Limited, a probiotic food company. The views expressed in the commentary are her own and not of the company.

## Authors' contributions

NH has put together the commentary after a thorough review of literature of the possible use of probiotics in the prevention of diarrhoea in the developing world.

GBN conceptualized the commentary and gave critical inputs related to the global incidence of diarrhoea, the need for interventions, and the possible utility of probiotics as a potential intervention.

NKG critically reviewed the commentary.

All authors have read and approved the final manuscript.
